# LimeSeg: a coarse-grained lipid membrane simulation for 3D image segmentation

**DOI:** 10.1186/s12859-018-2471-0

**Published:** 2019-01-03

**Authors:** Sarah Machado, Vincent Mercier, Nicolas Chiaruttini

**Affiliations:** 1 Marcos González Gaitán lab, University of Geneva, Department of Biochemistry, quai Ernest-Ansermet 30, Geneva, 1211 Switzerland; 2 Aurélien Roux lab, University of Geneva, Department of Biochemistry, quai Ernest-Ansermet 30, Geneva, 1211 Switzerland

**Keywords:** 3D segmentation, ImageJ, Surfel-based, Point-cloud, Cell volume, Cell surface, Cell membrane segmentation

## Abstract

**Background:**

3D segmentation is often a prerequisite for 3D object display and quantitative measurements. Yet existing voxel-based methods do not directly give information on the object surface or topology. As for spatially continuous approaches such as level-set, active contours and meshes, although providing surfaces and concise shape description, they are generally not suitable for multiple object segmentation and/or for objects with an irregular shape, which can hamper their adoption by bioimage analysts.

**Results:**

We developed LimeSeg, a computationally efficient and spatially continuous 3D segmentation method. LimeSeg is easy-to-use and can process many and/or highly convoluted objects. Based on the concept of SURFace ELements (“Surfels”), LimeSeg resembles a highly coarse-grained simulation of a lipid membrane in which a set of particles, analogous to lipid molecules, are attracted to local image maxima. The particles are self-generating and self-destructing thus providing the ability for the membrane to evolve towards the contour of the objects of interest.

The capabilities of LimeSeg: simultaneous segmentation of numerous non overlapping objects, segmentation of highly convoluted objects and robustness for big datasets are demonstrated on experimental use cases (epithelial cells, brain MRI and FIB-SEM dataset of cellular membrane system respectively).

**Conclusion:**

In conclusion, we implemented a new and efficient 3D surface reconstruction plugin adapted for various sources of images, which is deployed in the user-friendly and well-known ImageJ environment.

## Background

Over the recent years tremendous improvements have been made on the techniques allowing for acquisition of 3D images of biological samples at every scale. Volumetric datasets acquired by optical or electron microscopy, as well as with magnetic resonance imaging (MRI) broaden the scientific questions that can be investigated. The number of available bioimage analysis tools have risen accordingly. Image segmentation has a very long research history [1]. An inventory initiative accessible at http://biii.eu returns more than 1200 tools to date, which attests the interest and needs of such tools. Reviewing all methods is clearly beyond the scope of this work, but a few representative works and principles will be introduced to highlight LimeSeg specificities.

### Spatially discrete segmentation methods

A first set of commonly used segmentation methods are: intensity-based methods (simple thresholding, region-growing), mathematical morphology methods (watershed [2–4]) and various flavors of machine learning (from pixel classification [5, 6] to deep learning [7, 8]). These methods are all working in discrete space, their output being a label image in which every voxel is associated to a certain class or object (sometimes with a probability value). Segmenting many non overlapping objects naturally arises from the voxel-based nature of these methods since each voxel has a unique associated label. Another convenient property is that nothing particular is needed implementation-wise in order to segment objects with complex topology. However, voxel-based methods have some downsides. For instance, retrieving the surface of objects from a label image requires an extra processing step such as marching cubes [9] which is often deteriorating the smoothness of the shape and which could lead to bias [10]. It is also difficult to get sub-voxel properties, a feature that is very common in continuous methods (sub-diffraction spot localization, filament tracking [11, 12],...).

### Spatially continuous segmentation methods

A second set of commonly used segmentation methods are continuous based: snakes, level-set, active contours [13–20] or meshes [21, 22]. For these methods, no perimeter or surface reconstruction step is required. Continuous methods are mainly used to segment precisely or concisely the shape of a few objects (crawling cells, bones, animal...). Many continuous based methods are only suitable to segment a single object and are restricted to 2D images. Indeed the adaptation of these methods to multiple objects has potentially a high computational cost and requires complex algorithm adaptations [22–25]. Another shortcoming of methods such as 3D mesh based methods [21, 22] or snakes [26, 27] is that changes in object topology have to be taken into account explicitly in the implementation. Level-set methods do not experience this issue. Last, in snakes methods, due to the limited number of control parameters, the segmentation of tortuous objects is not possible. It is sometimes possible to take advantage of these restrictions to segment noisy images or to fit an object into a particular model [28, 29].

### LimeSeg

We present in this work a segmentation method which is loosely based on a molecular dynamics simulation of a lipid membrane. Each lipid is represented by an oriented particle, which is attracted by local image maxima. Lipids interact together to maintain the membrane (surface) integrity. Such an approach is intuitive and offers several advantages. It is a continuous method which is easy to implement. As with discrete methods, segmenting non overlapping objects is very easy, because implementing surface repulsion is straightforward. Moreover as lipids are loosely linked, topological changes occur naturally during the segmentation process, without explicitly taking these changes into account in the algorithm. Previous works based on a similar approach [30, 31], coined the term surfels (SURFace ELements) for these oriented particles. Oriented particle based systems have also been used for surface representation [32, 33], however an implicitly defined surface is also required in parallel of the particle system, contrary to our work. More generally, particle systems (not necessarily oriented) are extensively used for computer graphics and real time simulation [34]. However, to our knowledge, LimeSeg is the only work reporting a fully particle based surfel used for image segmentation and which is optimized enough to work on diverse and complex use cases.

## Principle

LimeSeg can be seen as a strongly coarse-grained modeling of a lipid membrane, where each “lipid” particle is attracted by the local underlying 3D image maxima. If lipids were only attracted to local maxima, no correlation would exist between lipid movement which could result in membrane dismantling. So, in LimeSeg as in the case of a real lipid membrane, each particle is also interacting with its neighboring lipids, in order to maintain the membrane integrity. However, unlike in a real physical system, we do not maintain the number of lipid constant. This allows the total surface to expand or shrink while adapting to the object being segmented. Thus, new particles are constantly added or removed to allow for surface adaptation, a process which is controlled through local particle density estimation. In practice, this density estimation is done by counting, for each particle, the number of particles included in a sphere of radius *d*_*threshold*_ centered on the particle of interest.

## Implementation overview

LimeSeg segmentation is an iterative process presented in Algorithm ??. Each iteration has three steps. In the first step the interactions between surfels is computed, the second step ensures surfel number adaptation according to surfel local density, in the third step the force exerted by the image on the surfels is taken into account and surfel position and orientation is updated accordingly.


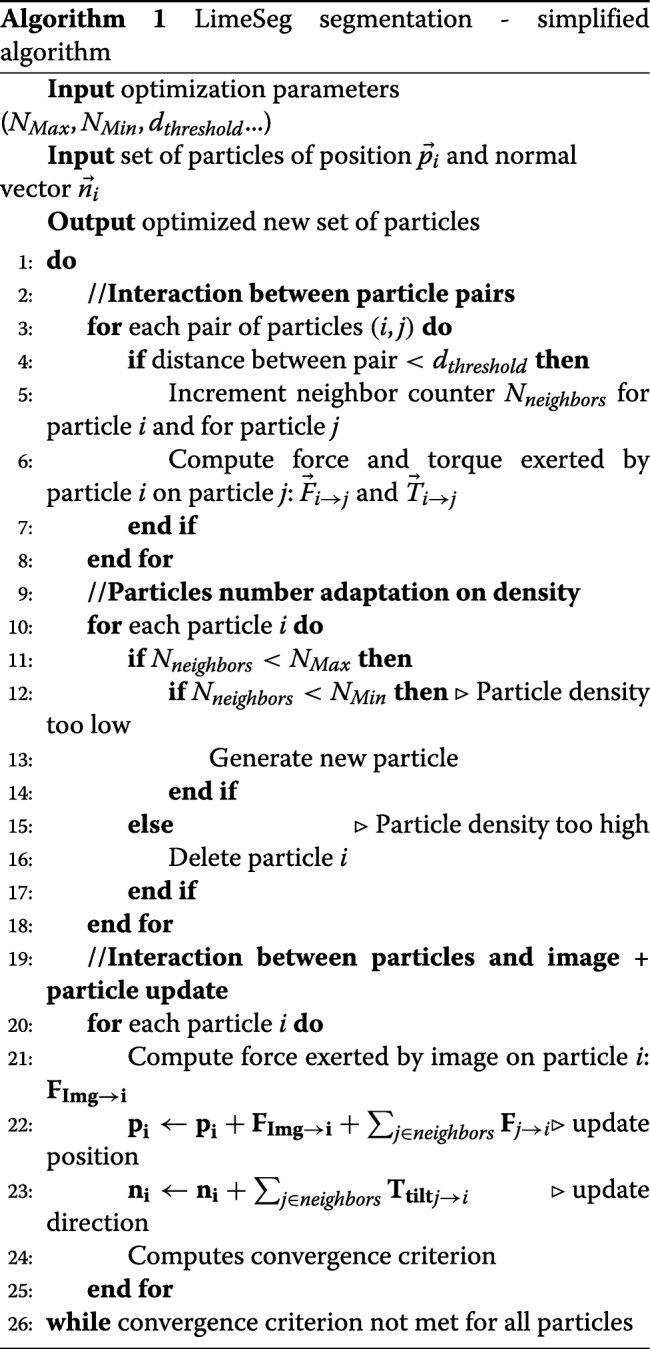


### Implementation details

**Fig. 1:**
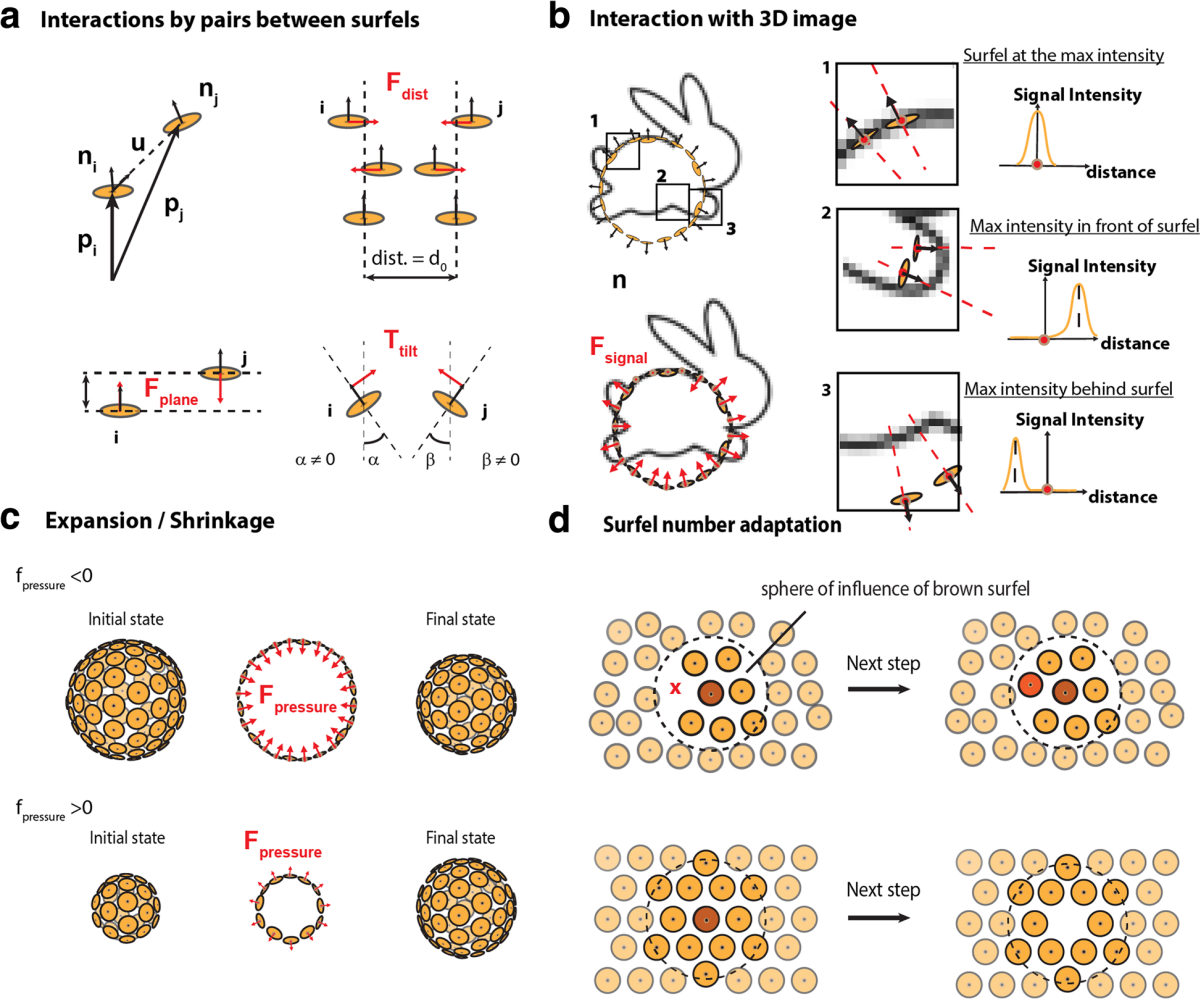
Surfel interaction rules. **a** - Forces and torque acting on a neighboring pair of surfel. Top left: notation convention for the position and normal vector of surfels. Top right: preferred distance interaction *d*_0_ and associated force **F**_**dist**_. Bottom left, planar interaction **F**_**plane**_. Bottom right, **T**_**tilt**_. **b** - Interaction with the 3D image. **F**_**signal**_ has a constant norm. It is positive, null, or negative depending on the local image maximum. **c** - **F**_**pressure**_ exerted along the normal vector. The sign of *f*_*pressure*_ controls surface shrinkage or expansion. **d** - Adaptation of surfel number depending on local neighbors. The number of neighbors within the sphere of influence is counted. Depending on this number, the surfel is removed or a new one is generated

Each surfel *i* is defined by a position in 3D: **p**_**i**_ and by a unit normal vector: **n**_**i**_ (Fig. 1a). The rules controlling the interactions between neighboring surfels were chosen based on the segmentation stability, consistency, reproducibility and speed, unlike more physically meaningful simulations [35–37]. The segmentation process starts from one or several seeds, each seed being a surfel system that delimits a surface. As detailed in the discussion, a seed is usually a sphere, but can also be a more complex shape made from a skeleton, or any pre-existing surfel system. The segmentation ends when all the surfels have converged, each iteration being divided into 6 main phases that are detailed below.

#### 1 - Neighboring surfel identification

Each surfel has a fixed-radius sphere of influence, and an equilibrium distance with his neighbors: *d*_0_. At this step, each surfel identifies and counts the number of surfels comprised within its sphere of influence. These surfels are considered as neighbors. The radius of the sphere of influence is by default *α*×*d*_0_ with *α*=1.75.

Setting a higher *α* leads to the computation of more interactions without noticeable advantage, and setting a lower *α* reduces the quality of the local density estimation, which is necessary for proper surfel number adaptation.

#### 2 - Neighboring surfel forces computation (Fig. 1a)

In agreement with previous works for oriented particle systems [30], we found that considering only pair interactions, short-range coupling with a few layers of neighboring surfels, and three interactions that are detailed below (**F**_**dist**_, **F**_**plane**_, **T**_**tilt**_) were sufficient to fulfill our requirements. Surfels interact with neighbors comprised through pair interactions. We note *d*=∥**p**_**j**_−**p**_**i**_∥ the distance and **u**=(**p**_**j**_−**p**_**i**_)/*d* the unit vector between surfels *i* and *j*. The first pair interaction $\textbf {F}_{\textbf {dist}_{j \rightarrow i}}=f(d/d_{0})\textbf {u}$ is the force that maintains the preferred distance *d*_0_ between pairs of surfels. If two surfels are separated by a distance smaller than *d*_0_, **F**_**dist**_ is a harmonic repulsive force. If the distance between the pair is above *d*_0_, **F**_**dist**_ mimics a bond that can break: it is attractive, vanishes at *d*=*d*_0_ and with *d*→*∞*. The second interaction $\textbf {F}_{\textbf {plane}_{j \rightarrow i}}=k_{plane}(\textbf {u}\cdot (\textbf {n}_{\textbf {i}}+\textbf {n}_{\textbf {j}}))\textbf {n}_{\textbf {i}}$ and third pair interaction $\textbf {T}_{\textbf {tilt}_{j \rightarrow i}}=k_{tilt}(\textbf {n}_{\textbf {i}}\cdot \textbf {u})\textbf {u}$ act on the position and on the surfel normal respectively. All together **F**_*j*→*i*_ ($=\textbf {F}_{\textbf {plane}_{j \rightarrow i}}+\textbf {F}_{\textbf {dist}_{j \rightarrow i}}$) and $\textbf {T}_{\textbf {tilt}_{j \rightarrow i}}$ are the interactions exerted by the surfel *j* on the surfel *i*. They both favor equal distance between particles and co-planarity.

#### 3 - Interaction with the image

Each surfel is attracted by the local underlying 3D image maximum **F**_**signal**_ and is biased by a constant pressure **F**_**pressure**_, allowing for surface adaptation to 3D objects contained in the image. First, **F**_**signal**_=±*f*_*signal*_**n** is the data attachment term of constant norm that links the particles to the 3D image. The direction of this force depends on the local image maximum location relatively to the normal vector (Fig. 1b). Second, **F**_**pressure**_=*f*_*pressure*_**n** is a fixed global pressure set by the user. This pressure tends to induce the shrinking or the expansion of the surface (Fig. 1c). It is equivalent to the “balloon force” used in related segmentation method [22, 38]. If the surfel is located near to a local image maximum, both **F**_**signal**_ and **F**_**pressure**_ become null.

#### 4 - Surfel number adaptation

During segmentation, the number of surfels needs to adapt: the number of surfel has to diminish while the surface shrinks and increase during surface expansion. For local surfel number adaptation, we implemented the following rules (Fig. 1d). 1) If the number of surfels comprised in its sphere of influence is higher than an upper limit, the surfel removes itself. 2) If the number is smaller or equal to the lower limit, a new surfel is created at the position of lowest surfel density. Practically, this position is estimated by computing the sum of the repulsive forces exerted by neighboring surfels. We have set up a balance period of a few iterations during which a newly created surfel can neither disappear nor generate a new neighboring surfel. We found that such a rule improves the stability of the system. Finally, to allow for clearance of spurious surface, surfels that are too isolated to create new surfels (because their number of neighbors is below the threshold) are removed.

#### 5 - Update of surfel position and orientation

The numerical integration follows an explicit Eulerian scheme combined to a purely viscous behavior: at each integration step, the displacement of each surfel is equal to its resulting force multiplied by *d*_0_: $\textbf {p}_{\textbf {i}}(t+1)=\textbf {p}_{\textbf {i}}(t)+d_{0}\times [\sum _{j\in neighbors} (\textbf {F}_{\textbf {dist}_{j \rightarrow i}}+\textbf {F}_{\textbf {plane}_{j \rightarrow i}})+\textbf {F}_{\textbf {pressure}}+\textbf {F}_{\textbf {signal}}]$, and the normal of each surfel is summed with the resulting sum of torques $\textbf {n}_{\textbf {i}}(t+1)=\textbf {n}_{\textbf {i}}(t)+\sum _{j\in neighbors} \textbf {T}_{\textbf {tilt}_{j \rightarrow i}}$. With this integration scheme, forces can be directly interpreted as displacement per integration step, in units of *d*_0_. For instance, if a constant force of value 0.01 is exerted on a surfel, and if *d*_0_ is set to 15 pixels, it will require 100 steps to move the surfel by 15 pixels.

#### 6 - Convergence test

The iterative process stops when all surfels are locked, as they met two convergence criteria. First, each surfel is considered as having converged when it undergoes little displacement or rotation in the course of a defined number of integration steps. Second, when all its neighbors have converged, the position and normal vector of a defined surfel are locked. The above two-step convergence can be used to restrict the active computation zone and speed up thce segmentation (see FIB-SEM segmentation part).

### Implementation efficiency

Of the different phases of the optimisation loop, neighboring surfels identification, (also called fixed-radius near neighbor search problem), is the most computationally intensive. To accelerate this step, we implemented a custom space-partitioning tree building algorithm, which is further parallelized on graphics processing units (GPU) using CUDA library and the Java JCUDA wrapper. The computation of pairs of forces is also a time consuming step which can also be processed on GPU. Nonetheless, CPU computation remains faster for low number of particles, thus LimeSeg automatically switches between CPU and GPU with a threshold at 20k surfels. Overall, with these optimizations, an integration step scales almost linearly with the number of particles (*N*^1.05^) and three parts (1 - 2 - 3) contributes almost equally to 90% of the integration time.

## Results

We first review the emergent properties of the simulated set of particles. LimeSeg is controlled by two sets of parameters: i) parameters ruling the particle system (*α*, *f*(*d*/*d*_0_), *k*_*plane*_, *k*_*tilt*_, *f*_*pressure*_, density thresholds and convergence criteria), ii) parameters ruling the interaction of particles with the image (*d*_0_, *f*_*signal*_, band width over which a maximum is looked for). Not all parameter combinations are appropriate. Some combinations lead to particle instability or to particles that ignore the image influence. By trials and errors, we found a set point in the phase space of parameters which allows for a very good stability of the system, while keeping the surface ability to be deformed under the image influence. In LimeSeg, all parameters are set by default to values matching this set point except two: *d*_0_ and *f*_*pressure*_.

- *d*_0_ is the equilibrium distance between surfels, expressed in number of pixels. It is the most essential parameter of LimeSeg as it sets the minimum feature size that can be segmented. In typical use cases, this value lies between 1 and 20 pixels.
- *f*_*pressure*_ is the force biasing the surface movement towards inflation (positive) or deflation (negative). Like any other force within LimeSeg it has the unit of a distance per integration step, in units of *d*_0_. It should lie between −0.04 and 0.04 to keep the particle system stable.

These two key parameters should be set by the user according to its use case. We show in the following section how the system behaves in synthetic test cases and demonstrate how these two parameters can be modified to bring the particle system to an expected behavior.

### Leakage / Arrest

**Fig. 2:**
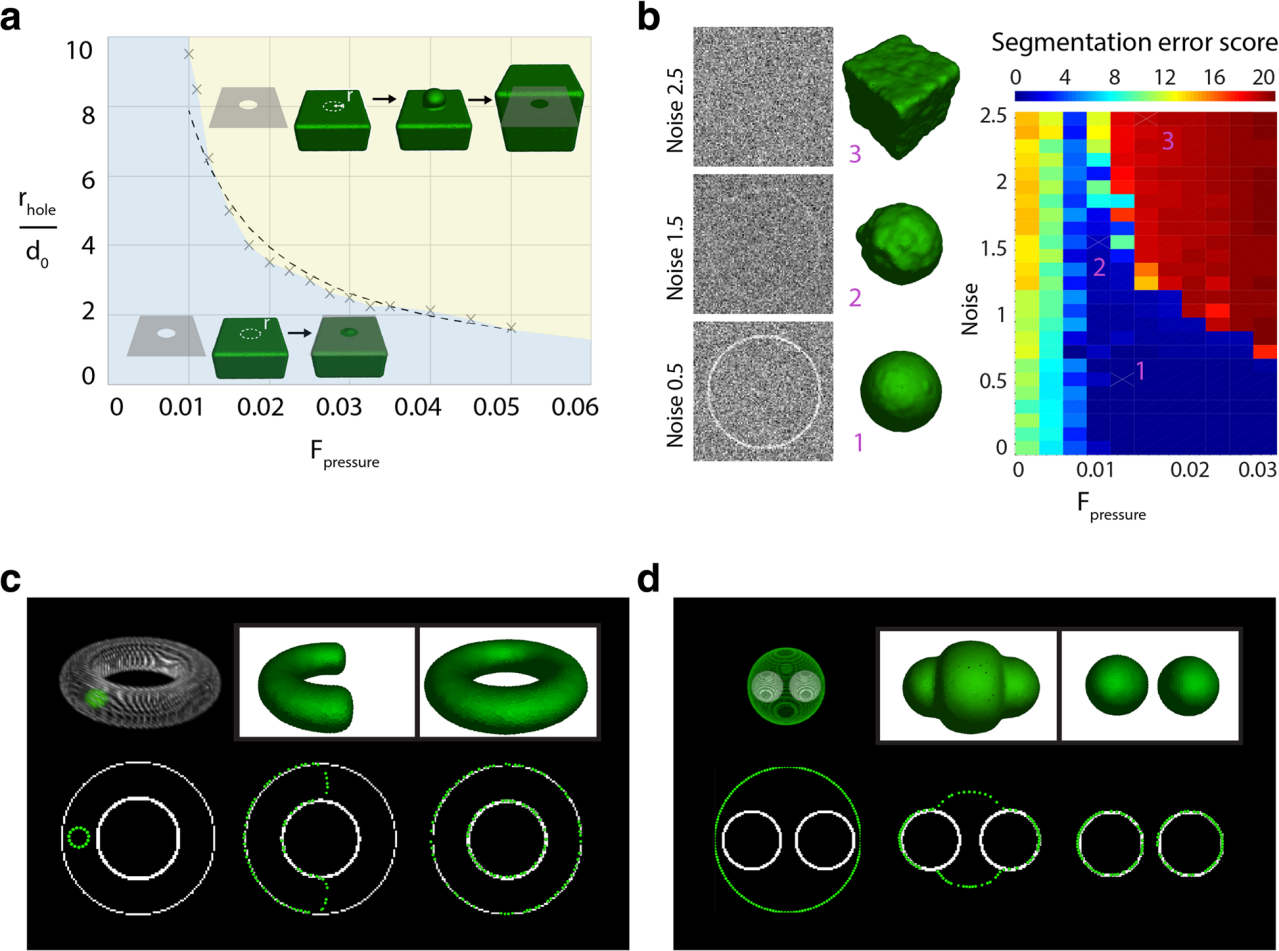
Point cloud mechanics characterization. **a** - Behavior of surfels network with fixed *d*_0_ as a function of *f*_*pressure*_ when it encounters a circular hole of radius *r*_*hole*_. In the blue region, the surfel mesh does not cross the hole. In the yellow region the surfel mesh flows through the hole. A 1/r dotted line approximates the frontier between these regions. **b** - Image noise segmentation benchmark, see text for details. Left: equatorial plane of sphere image with various noises and resulting segmentation. Right: Segmentation score (i.e. root mean square of surfel distance to the target sphere in pixel) as a function as noise and *f*_*pressure*_, all other parameters are unchanged. **c** - Surface fusion test. The initial state consists of a spherical seed inside a torus. After several iterations, the shape of the segmentation surface successfully merges with itself (*f*_*pressure*_>0). **d** - Surface fission test. The initial state consists of a spherical seed surrounding two spherical objects. The segmentation surface successfully splits during the course of the segmentation (*f*_*pressure*_<0)

A common problem to overcome in segmentation is leakage: the surfel surface could improperly spread through small “holes” where the data outline is missing or weak. As a result, voxels that not do belong to the object would be included into the object. Conversely, little holes could also be part of the original object (the beginning of a “neck” or of a tube for instance). In that case, the surfel system should go through the hole. Indeed, a surface that would stop around the hole would converge without reaching the outline of the object, resulting in an incomplete segmentation. Depending on the parameters chosen by the user, both behaviors can be obtained with LimeSeg. As a demonstration, we segmented a test case consisting of a 100x100x100 image cut in half by a plane containing a circular hole of radius *r*_*hole*_ in its center. We segmented this image while varying the radius of the hole *r*_*hole*_ and *f*_*pressure*_ but keeping *d*_0_ constant. Depending on the parameter combination, two outputs are observed: i) the surface stops around the hole (required in the case of artifactual holes in the image signal) or ii) the surface goes through the hole and continues growing (required to segment an object containing a tube, a neck) (Fig. 2a). In a diagram plotting *r*_*hole*_/*d*_0_ as a function of *f*_*pressure*_, these two segmentation outcomes are found in two distinct domains separated by a 1/r curve. In other terms, the frontier is governed by an intrinsic quantity, which is equal to the radius multiplied by the pressure. This quantity has the unit of a surface tension, and reveals an intrinsic emerging threshold of the system. It can be understood as follows: during segmentation, the radius of the hole combined with the applied pressure sets transiently a surface tension that can be computed by the Young-Laplace equation and that is withheld by the particle network. If the tension is above the threshold, the link between surfels are disrupted and new surfels are generated to fill the gaps, leading to expansion of the surface. If the tension is below the threshold, surfel interactions are maintained, the surface is stable and the convergence can be reached without going through the hole. In conclusion, the user can tune *f*_*pressure*_ and *d*_0_ to adapt the segmentation to the required output. Intuitively, lowering *d*_0_ or increasing *f*_*pressure*_ leads to a better penetration of the surface through gaps.

### Noise resilience

Another common matter of interest in segmentation is the method resilience to image noise. We address in a simple test case how the signal to noise ratio affects the segmentation outcome. Starting from a slightly off-centered spherical seed, we segment a larger sphere while varying the noise contained in the image. In our test image, the edge of the sphere has on average 2 pixels in thickness, with a variability depending on the 3D rasterization and a signal value of 1. A Gaussian noise centered on zero was added on the image, with standard deviation values ranging from 0 to 2.5 (typical lateral slices are shown Fig. 2b, left). We evaluated the segmentation outcome with positive *f*_*pressure*_ values varying from 0.005 to 0.035. To evaluate the reliability of the segmentation, we measured after convergence, the deviation of the distance from each particle to the target sphere (Fig. 2b, right). When the intensity of the positive pressure was too low (<0.01), even a very small noise was preventing the seed inflation,which lead to incorrect segmentation. Conversely, the seed could pass the outline of the sphere on a signal to noise ratio dependent manner, in the case of high positive pressures (>0.03). There is an optimal value for *f*_*pressure*_ around 0.01, which allows for the object outline detection at a low signal to noise ratio. For noisy images, a *f*_*pressure*_ value around 0.01 is thus recommended.

### Surface topology

Another relevant information about a segmentation method is how it handles topological changes, i.e. can a surface spontaneously split and merge? To test LimeSeg for intrinsic merging, we segmented a torus starting from a spherical seed located inside the torus. We used a positive pressure and observe that the two ends of the “C” shape are fusing to form the torus (Fig. 2c). To test for fission, we segmented two spheres starting from one unique spherical seed, which was surrounding the two target spheres. We applied a negative pressure and starting from one seed we obtained two distinct segmented surfaces (Fig. 2d). In conclusion, LimeSeg handles topological changes such as fusion and fission. As already explained in the introduction and in contrast with mesh methods, the topological changes naturally arise from the particle set interaction rules.

## Discussion

We demonstrate through several use cases the capabilities and versatility of LimeSeg. The imaging modalities (confocal microscopy, MRI, FIB SEM), image contrast and resolution, signal to noise ratio, shape size and object density are also different in these examples. Through these examples, features of LimeSeg are exemplified such as the 3D segmentation of big objects (15 millions surfels for the cell membrane system), of highly convoluted objects (brain / cell membranes) and of multiple spatially excluding objects (cells of an epithelium).

### Segmentation of lipid vesicles

**Fig. 3:**
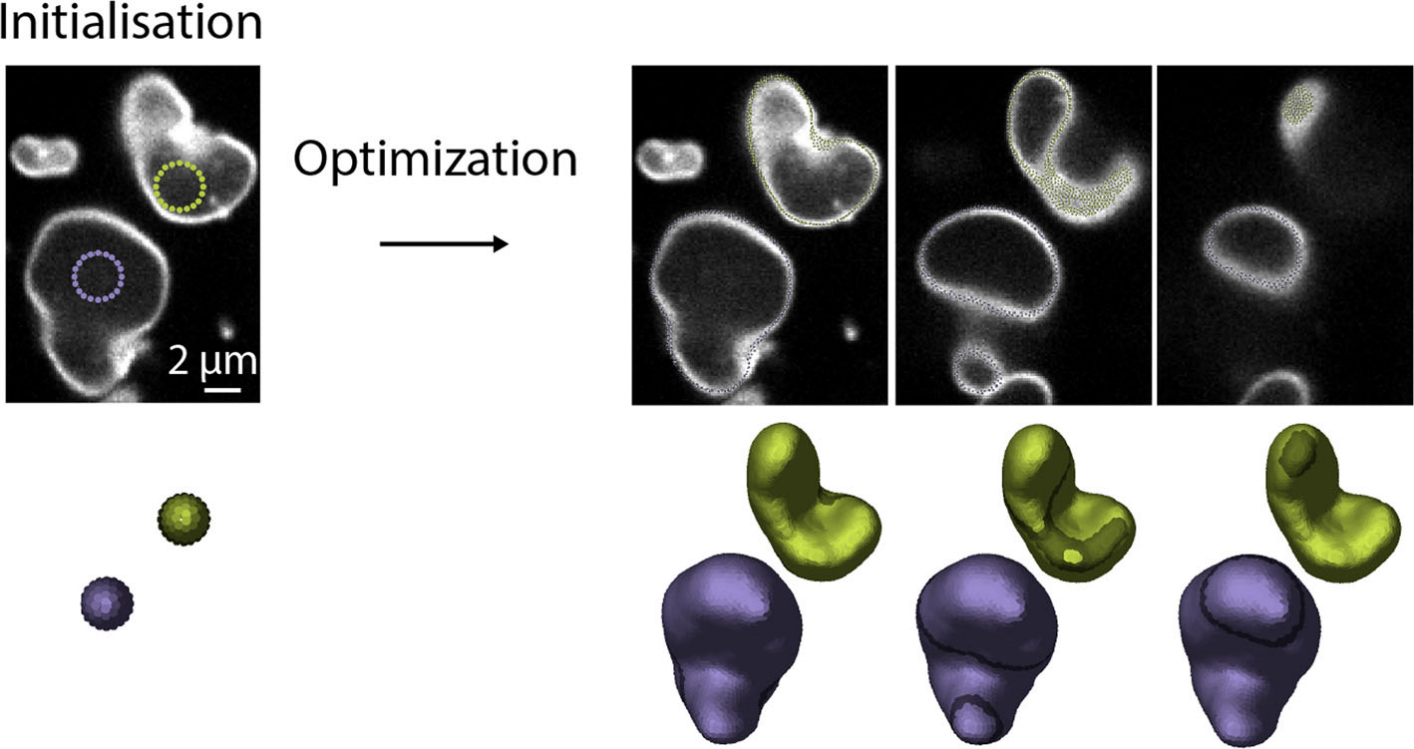
Segmentation of deformed lipid vesicles. The two vesicles are segmented sequentially. Right: segmentation outcome. Three z slices where surfels appear as dots are shown as well as the 3D reconstruction, where the in-planes surfels are highlighted

This first basic test consists in segmenting the surface of deformed lipid vesicles, which are attached on a glass coverslip. The vesicles are imaged with a confocal microscope that outputs a 3D image stack. We segmented two vesicles sequentially, starting from spherical seeds located inside each vesicle. We show in Fig. 3 how the point set matches the outlines of these two vesicles. Each vesicle segmentation takes a few seconds and each vesicle is made of approximately 1000 particles.

### Segmentation from MRI images: full human brain segmentation

**Fig. 4:**
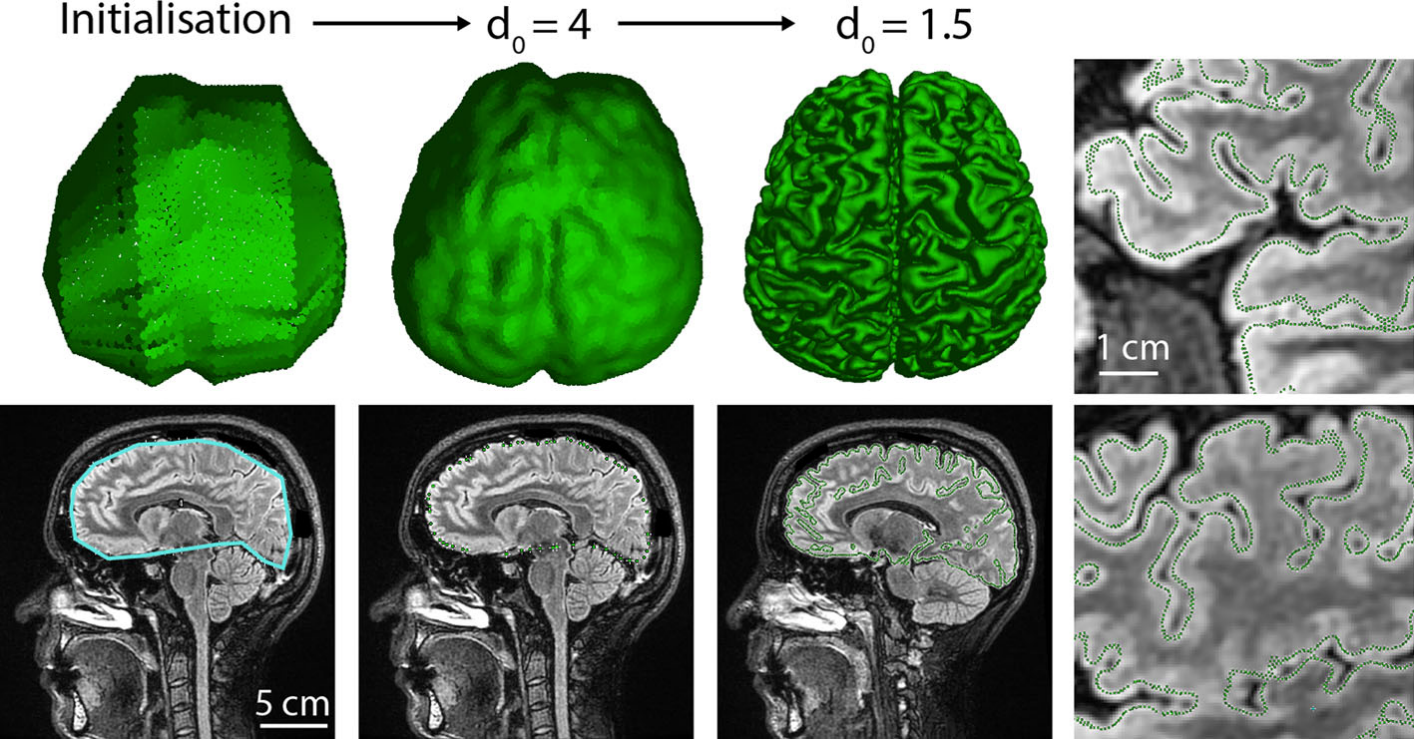
Human brain MRI surface segmentation. From left to right: 1 - initialization of the shape with ROI skeleton (blue line on the data image). 2 - After segmentation convergence with *d*_0_=4, many details of the cortex are missed. 3 - Segmentation refinement by decreasing *d*_0_ to 1.5. 4 - Zooms showing details being retrieved by the finest segmentation where surfels appear as green dots

In this test case, we segment the cortical surface of an MRI dataset (FLAIR sequence, see Fig. 4, bottom), which consists of 512x512x224 voxels. We set an initial “skeleton” seed which is slightly larger than the brain. This skeleton (or non-spherical seed) is used to initialize the segmentation and consists of roughly defined ROIs surrounding the brain at specific slices through the stack (Fig. 4, left). Using the user specified *d*_0_ value, the plugin can dispatch surfels on this basic geometrical skeleton before starting the segmentation. When convergence is reached with *d*_0_=4, one can notice that finer details of the cortex are missed (see for instance the blue region of Fig. 2a). It indicates that the size of brain convolutions is too small relatively to *f*_*pressure*_ and *d*_0_. In such a case, the segmentation process can be refined by progressively reducing *d*_0_. In this example, we refined the brain segmentation by reducing *d*_0_ down to 1.5 pixels and resumed the segmentation to reach final convergence. At the end of the segmentation, the fine brain convolutions are detected (Fig. 4, right). The whole process took 5 min and resulted in a 300,000 point cloud.

### Plasma membrane and endoplasmic reticulum segmentation

**Fig. 5:**
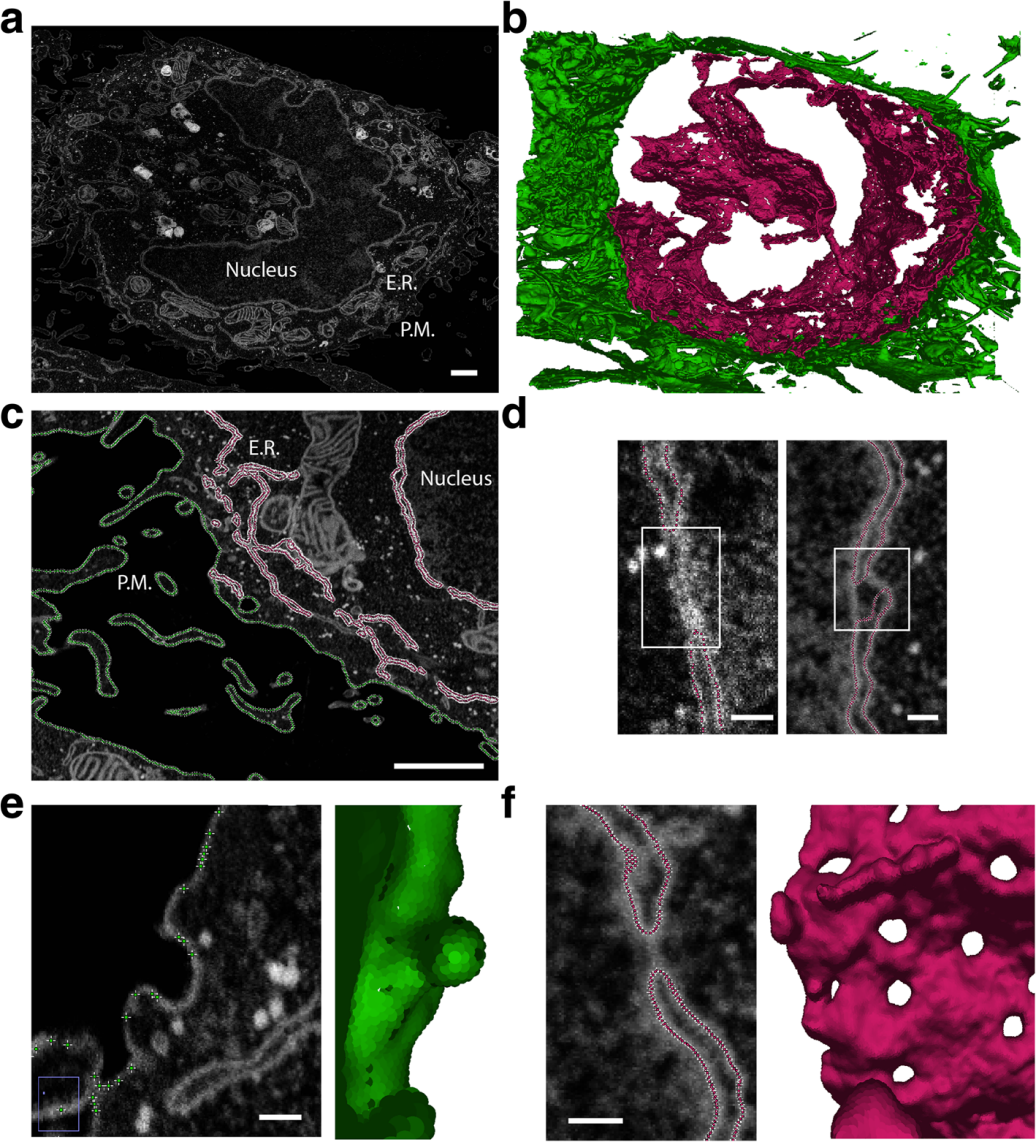
Endoplasmic reticulum (ER) and plasma membrane (PM) segmentation of a FIB-SEM HeLa cell dataset. **a** - Typical data slice where the nucleus, ER and PM are visible. **b** - Resulting segmentation of ER (magenta) and PM (green). **c** - Segmented ER and PM, overlaid on the original data. **d** - Missed parts of nuclear envelope where the double membrane is too thin to be correctly segmented (left). Spurious hole generated during segmentation (right). **e** - Detail showing plasma membrane invagination in 2D and 3D. **F** - Detail of nuclear pore complex as seen on 2D and on 3D. Scalebars: **a**, **c**: 1*μm*; **d**, **e**, **f**: 100*nm*

We challenged the method by segmenting a 3D EM dataset of 4136x3120x626 voxels. The dataset consists of nearly isotropic sections of a Hela cell (4.13 nm in XY, 5 nm in Z) (Fig. 5a) prealigned with TrackEm2 [39], without additional preprocessing. We aimed to segment two structures sequentially: the plasma membrane and the endoplasmic reticulum (ER). For such a big dataset, it is expected that during the course of the segmentation, a large portion of surfels will have converged, while relatively small regions will continue to grow actively. Keeping all the points that have converged at each integration step induces unnecessary computational cost. To circumvent this problem, LimeSeg, like other segmentation methods [17], has a way to restrict the computation to actively segmenting regions. In brief, while constructing the space partitioning tree, LimeSeg detects and replace large chunks of locked surfels by a single super surfel. The conversion of active surfels into passive and locked super surfels is reversible. If a particle that is not locked is interacting with a super surfel, the super surfel is replaced by the chunk of surfels it contains in the next integration step, allowing for rearrangements.

The plasma membrane segmentation was performed starting with 5 spherical seeds located outside of the cell. The seeds have inflated and merged until they have surrounded the cell. This segmentation took 4 h on a standard desktop computer with an entry-level graphic card and resulted in a 4 million particle point cloud (Fig. 5b, c, green). We next segmented the ER system. We initiated the segmentation with 5 spherical seeds located into the lumen of the ER and ran 80,000 integration steps over 6 h. This led to a cloud of 15 million points in which the double nuclear envelope, that is inherently linked to the ER system, was segmented as well (Fig. 5b, c, magenta). Some limitations can be seen: inexistent holes are sometimes detected and the ER cannot be segmented when two membranes are too close (ER lumen too thin, Fig. 5d). We believe that this segmentation is still very satisfactory given the very little amount of work required by the user. Many aspects of the cell membrane geometry are preserved and can be detected in the segmentation: membrane invaginations like clathrin coated pits (Fig. 5e), nuclear pore complexes (Fig. 5f), the complex network of intertwined filopodia and the highly convoluted ER shape (Fig. 5c). Thus, the segmentation generated with LimeSeg provides a very good starting point for further shape analysis, like proper surface quantification and curvature measurements.

### Cell segmentation and cell volume measurement form confocal images: the case of a Drosophila egg chamber

**Fig. 6:**
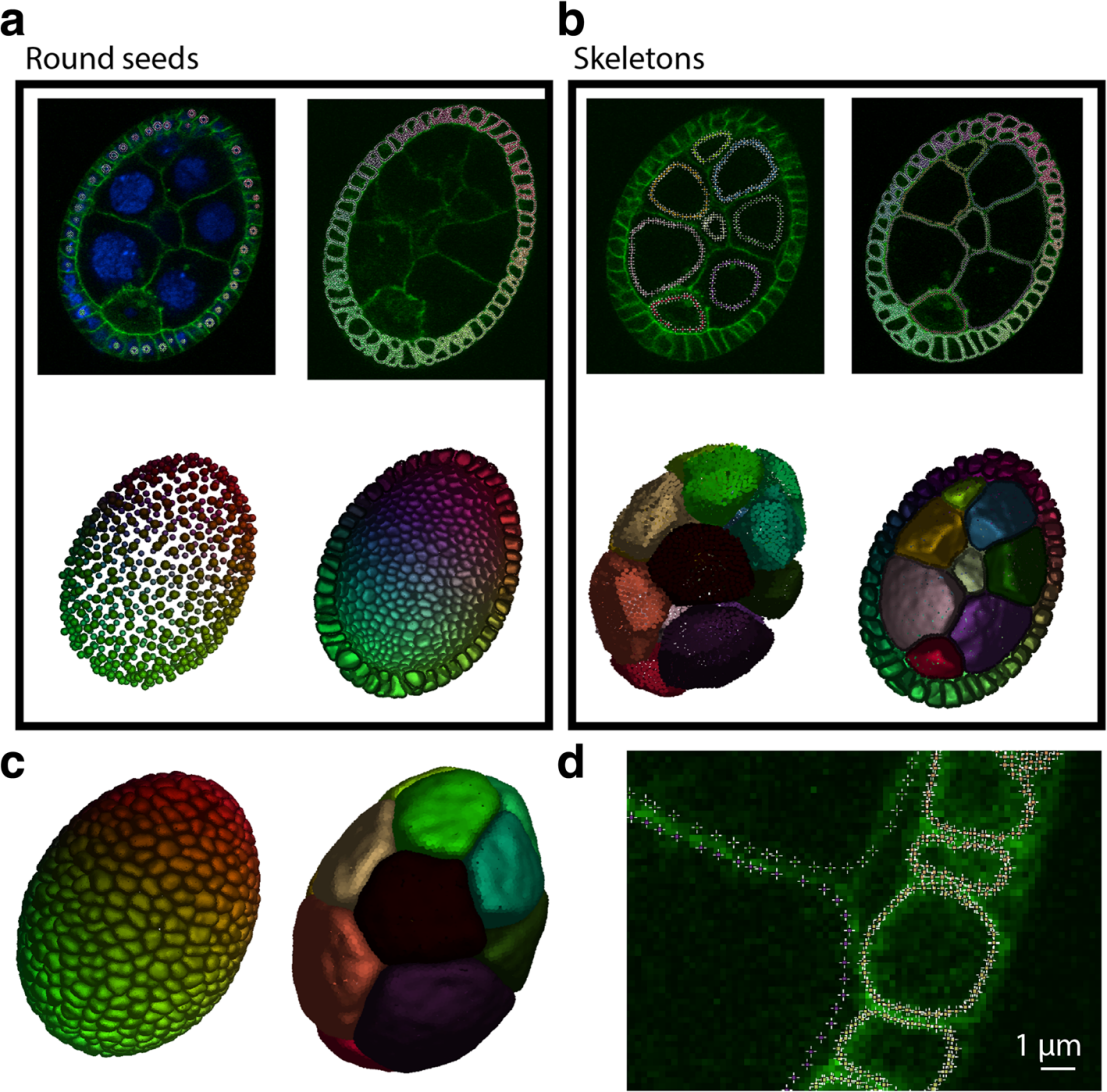
Drosophila egg chamber segmentation. **a** - Segmentation of the follicle cells. Up: surfels appear as colored dot (one color per cell). Below: 3D reconstruction. Only the surfels below the shown slice on top are represented. Left: initial state; right: final state. **b** – Segmentation of the nurse cells. During the course of segmentation, surfels of follicle cells were locked to maintain the egg outline. **c** – 3D reconstruction output for nurse cells and follicle cells. **d** – Detail of surfel positions after segmentation convergence

In the previous examples, only one object is being segmented at a time. We show with this example that multiple objects can be segmented simultaneously by delimiting cells from confocal fluorescent slices of a drosophila egg chamber (Fig. 6a). The egg chamber is an interesting case study as it consists of three different cell types which shape and size are very different: nurse cells, follicle cells and the oocyte. As a prerequisite for segmentation, the user needs to provide LimeSeg the approximate position of each cell. This seeding can be done in many ways: manually, by identifying local minima in a blurred image, by using the fluorescent channel of nuclei (like we did) and by computing the barycenter of each nuclear blob. At each position, a sphere of predefined radius serves as an initial point set. The user then specifies that each sphere is a different object and then LimeSeg sets a unique identifier to all surfels of a particular cell spherical seed. This part is in contrast with the FIB-SEM dataset, where each sphere was attributed to a unique object identifier. Based on these identifiers, surfel-surfel interactions are differentiated. If two interacting surfels belong to the same cell, the interactions are as described before. Conversely, if two surfels of different cells interact, they are not considered as neighbors and only the repulsive part of **F**_**dist**_ is kept, allowing for surface repulsion.

The egg chamber segmentation was carried out in two stages. We first segmented the follicle cells using a low value for *d*_0_, necessary to resolve the geometry of these small cells (computation time 5 min) (Fig. 6a). Then we locked this set of points, which defines the egg chamber periphery. Second, we initialized the segmentation of the oocyte and of the nurse cells using skeleton seeds and a higher *d*_0_ value (Fig. 6b, left). These bigger cells are segmented while keeping fixed points of the follicle cells to maintain the outline (Fig. 6b, right). We show in Fig. 6c the segmentation result for these two types of cells and some details of surfel positioning in the image in Fig. 6d. The processing of this example image took 10 min and resulted in a cloud of 380,000 points, which can be used for further quantifications.

### Cell tracking and shape analysis of LimeSeg outputs

Even if not shown in this manuscript, LimeSeg supports analysis of time series and multichannel images. In particular, for limited object shape changes between successive frames, the object shape can be segmented over time by providing the output of the previous frame as an input to the following frame.

A point cloud is the structure used during segmentation, but it is not the best structure objects to perform further object shape analysis. A polygonal mesh is much more suited. LimeSeg provides a surface reconstruction algorithm from its point cloud and functions for basic shape analysis (volume / surface / surface center of mass). The data can be accessed directly via ImageJ commands, or via scripting. As an alternative to ImageJ, the point cloud and/or the meshes can also be exported in the standard ply file format, which then can be imported into other software for further processing and analysis.

## Conclusion

In conclusion, we implemented a new surface reconstruction plugin adapted for various sources of images. LimeSeg is intended to be used to segment one or multiple objects in a modular fashion. It has been optimized to enable segmentation of relatively large images, using graphical processing units for the most consuming time steps and the generic ImgLib2 library [40]. To facilitate the work of bio-image analysts, LimeSeg is implemented as an ImageJ / Fiji [41–43] plugin, a software which is under very active development and with which the microscopy and image analysis community are already familiar with. LimeSeg user interface is composed of a recordable graphical user interface GUI, an ImageJ application programming interface, and provides a 3D viewer. On the user interface side, it can be used with simple predefined commands that require initial seeds and 2 parameters. More detailed instructions regarding the software usage, customization, tutorials and updates are available on the ImageJ wiki (https://imagej.net/LimeSeg). The plugin is available via its ImageJ update site (http://sites.imagej.net/LimeSeg/) and the code is available on GitHub (https://github.com/NicoKiaru/LimeSeg).

## Methods

Experimental datasets used in this study:

- Vesicles are giant unilamellar vesicles made of DOPC, supplemented with 0.1% DOPE-Atto647N (ref AD-647N, Atto-tec, Germany) and 0.03% DSPE-PEG(2000) Biotin (ref 880129, Avanti Polar Lipids, USA) electroformed during 1 h at 1V RMS [44] in a sucrose buffer at 250 milliosmoles. Vesicules were adhered on avidin coated glass coverslips, deflated with an hyperosomotic shock due to buffer evaporation and imaged with a Yokogawa spinning-disc CSU-X1 mounted on a Nikon Ti-Eclipse microscope stand using a 100x objective with NA 1.3 (z spacing 340 nm, xy pixel size 122 nm).
- MRI dataset was acquired from a normal healthy person, using a FLAIR sequence.
- FIB-SEM 80% confluent HeLa cells were rinsed once with PBS, fixed for 3h on ice using 2.5% glutaraldehyde/2% paraformaldehyde in buffer A (0.15M cacodylate, 2mM CaCl2). Then cells were extensively washed on ice in buffer A, pelleted and incubated 1h on ice in 2% osmium tetroxide and 1.5% potassium Ferro cyanide in buffer A and finally rinsed 5 times in distilled water at room temperature. Cells were then incubated 20min at room temperature in 0.1M thiocarbohydrazide, which had been passed through a 0.22 *μ*m filter, and extensively washed with water. Samples were incubated overnight at 4° C protected from light in 1% uranyl-acetate, washed in water, further incubated in 20mM lead aspartame for 30min at 60°C and finally washed in water. Samples were dehydrated in a graded series ethanol, embedded in hard Epon and incubated for 60h at 45°C then for 60 h at 60°C. A small bloc was cut and mounted on a pin, coated with gold and inserted into the chamber the HELIOS 660 Nanolab DualBeam SEM/FIB microscope (FEI Company, Eindhoven, Netherlands). ROI were prepared using focused ion beam (FIB) and ROI set to be approximatively 20 microns wide. For imaging, electrons were detected using Elstar In-Column secondary electrons Detector (ICD). During acquisition process, the thickness of the FIB slice between each image acquisition was 5 nm.
- The drosophila egg chamber is dissected from a drosophila ovary. Cell nuclei were stained with DAPI and cell membranes labeled with the fusion proteins Nrg::GFP and Bsg::GFP [45]. The egg chamber was embedded in Vectashield and spacers were used to prevent tissue deformation. Images were acquired using an inverted Olympus point scanning confocal microscope IX81 with a 60x objective NA 1.42(z spacing 750 nm, xy pixel size 265 nm).

## Availability and requirements

**Project name:** LimeSeg

**Project home page:** https://github.com/NicoKiaru/LimeSeg

**Operating system(s):** Platform independent

**Programming language:** Java

**Requirements:** ImageJ/Fiji

**License:** CC0 Public Domain
